# Supplementary material for: The global distribution of the arbovirus vectors Aedes aegypti and Ae. albopictus
Source: eLife. 2015 Jun 30;4:e08347. doi: 10.7554/eLife.08347 (PMC4493616; doi:10.7554/eLife.08347)
Supplement: Supplementary file 1. — List of contributors and their affiliation from TigerMaps & VBORNET for Ae. albopictus presence records in Europe. DOI: http://dx.doi.org/10.7554/eLife.08347.012 [file elife08347s001.docx]

Supplementary file 1: List of contributors and their affiliation from TigerMaps & VBORNET for *Ae. albopictus* presence records in Europe.

**Albania:** Bino S. & Dikolli Velo E., Institute of Public Health, Tirana.

**Austria:** Allerberger F., Hufnagl P., Austrian Agency for Health and Food Safety, Vienna; Seidel B., Technical Office of Ecology and Landscape Assessment, Persenbeug.

**Bulgaria:** Mikov O., National Centre of Infectious and Parasitic Diseases, Sofia.

**Croatia:** Merdic E., Klobucar A., Department of Biology, University of Osijek.

**Czech Republic:** Hubalek Z., Institute of Vertebrate Biology, Brno.

**France:** L’Ambert G., Jeannin C. & Perrin Y., Adege, EID Méditerranée, Montpellier, France

**Germany:** Kampen H., Friedrich-Loeffler-Institut, Greifswald - Insel Riems; Kuhn C., Umweltbundesamt (Federal Environment Agency), Berlin; Pluskota B., German mosquito control association (KABS), Waldsee.

**Greece:** Gewehr S., Ecodevelopment S.A., Thessaloniki; Koliopoulos G., Benaki Phytopathological Institute, Laboratory of Biological Control of Pesticide, Kifissia; Voutsina N., Centre for Mosquito Abatement and Civic Protection, Prefecture of Serres.

**Italy:** Abbona I., ASS n1 Triestina; Albonetti P., AUSL Genova; Alessio A., AUSL Citta di Bologna; Albieri A. & Bellini R., CAA, Crevalcore; Angelini P., Servizio di Sanità Pubblica, Regione Emilia-Romagna, Bologna; Battistini G., AUSL, Parma; Biasci A., Entomox Pisa; Baldaccini G., ARPAT Toscana; Cafiero M.A., IZS delle Puglie; Caprioglio A., Regione Piemonte; Calzolari M., ISZLER, Reggio Emilia; Carasi S., AUSL, Brescia; Casaletti G., AUSL, Modena; Casarini P., ARPA Lombardia, Pavia; Chiatante A., AUSL, Piacenza; Dalla Pozza G., AUSL 16, Venezia; Di Domenicantonio R., Dept. Comune di Roma ; Drago A. & Martini S., Entostudio Padova, Universita di Padova; Fabbri C., AUSL Ravenna; Frilli F., Ist. Protez. Piante, Universitá di Udine; Gavaudan S., Istituto Zooprofilattico Sperimentale Umbria e Marche; Giangaspero A., Universita di Foggia; Giannetto S. & Brianti E., Universita di Messina; Grelloni V., IZS dell’Umbria; Mascali Zeo S., Baldacchini F., AUSL Cesena¸Massi R, Machiodi G., AUSL Bergamo; Mosca A., Ipla SpA, Torino; Otranto D. & Lia R., Universita di Bari; Pinna G., CPAI Cagliari; Raineri V., APAT Genova; Romi R. & Severini F., Istituto Superiore di Sanità, Roma; Scarpellini P. AUSL Forlí; Roiz D., Centro de Ecologia Alpina-Fundazione Edmund Mach, Trento; Talbalaghi A., Mosquito Control Piemonte, Alessandria; Tamburro A., Cocchi M., Pontuale G., AUSL 9, Grosseto and Regione Toscana; Venturelli C., Dipartimento di Sanita Publica AUSL, Cesena; Zamburlini R., Ist. Protez. Piante, Universita di Udine.

**Malta:** Gatt P., University of Malta.

**Netherlands:** Ibáñez Justicia A. & Scholte E.J., National Centre for Monitoring of Vectors, Wageningen.

**Romania:** Faculta E. & Prioteasa F.L., National Institute of Research-Development for Microbiology and Immunology "Cantacuzino", Bucarest.

**Russia:** Sergiev V.P., Martsinovsky Institute of Medical Parasitology and Tropical Medicine, Moscow

**Serbia:** Petrić D., Faculty of Agriculture, Novi Sad.

**Slovenia:** Kalan K., University of Primorska, Koper.

**Spain:** Aranda C., Consell Comarcal del Baix Llobregat, Sant Feliu de Llobregat; Bueno-Mari R., Cavanilles Institute of Biodiversity and Evolutionary Biology, University of Valencia, Paterna; Eritja R., Consell Comarcal del Baix Llobregat, Sant Feliu de Llobregat; Escosa R., CODE, Amposta; Lucientes J., Dept. Parasitología, Fac. de Veterinaria, Univ. de Zaragoza, Zaragoza; Marquès E., Servei de Control dels Mosquits de la Badia de Roses i Baix Ter, Castello d'Empuries; Miranda M.A., Universitat de les Illes Balears, Palma de Mallorca; Sánchez López P.F., Health and Consumers Department of the Government of La Región de Murcia.

**Switzerland:** Flacio E., Istituto Cantonale di Microbiologia, Bellinzona; Schaffner F., Institute of Parasitology, University of Zurich, Zurich, Switzerland

**Turkey:** Alten B., Hacettepe University, Ankara.
